# Development and validation of a new standardized measure for assessing experiences of discrimination within mental health services. A participatory research project

**DOI:** 10.1017/S2045796023000689

**Published:** 2023-09-08

**Authors:** Antonio Lasalvia, Stefano Pillan, Giulia Marzocco, Anna Ambrosini, Franco Veltro, Tecla Pozzan, Camilla D’Astore, Doriana Cristofalo, Mirella Ruggeri, Chiara Bonetto, the Mental Health Service Users’ Association ‘Il Cerchio Aperto’

**Affiliations:** 1 Section of Psychiatry, Department of Neurosciences, Biomedicine and Movement Sciences, University of Verona, Verona, Italy; 2 UOC Psichiatria, Azienda Ospedaliera Universitaria Integrata (AOUI) di Verona, Verona, Italy; 3 Mental Health Department of Campobasso, Campobasso, Italy; 4 Associazione Italiana Diffusione Interventi Psicoeducativi in Salute Mentale (AIDIPSaM – APS), Italy

**Keywords:** discrimination, healthcare providers, mental health services, psychometric validation, stigma

## Abstract

**Aims:**

People with mental disorders frequently report experiences of discrimination within mental health services, which can have significant detrimental effects on individuals’ well-being and recovery. This study aimed to develop and validate a new standardized measure aiming to assess experiences of stigmatization among people with mental disorders within mental health services.

**Methods:**

The scale was developed in Italian and tested for ease of use, comprehension, acceptability, relevance of items and response options within focus group session. A cross-sectional validation survey was conducted among mental health service users in Italy. Exploratory factor analysis with Promax oblique rotation, the Kaiser**–**Meyer**–**Olkin (KMO) measure of sampling adequacy and the Bartlett’s test of sphericity were used to assess the suitability of the sample for factor analysis. Reliability was assessed as internal consistency using Cronbach’s alpha and as test–retest reliability using weighted kappa and intraclass correlation coefficient (ICC). Precision was examined by Kendall’s tau-b coefficient.

**Results:**

Overall, 240 people with mental disorders participated in the study; 56 also completed the retest evaluation after 2 weeks. The 18 items of the scale converged over a two-factor solution (‘Dignity violation and personhood devaluation’ and ‘Perceived life restrictions and social exclusion’), accounting for 56.4% of the variance (KMO 0.903; Bartlett’s test *p* < 0.001). Cronbach’s alpha for the total score was 0.934. The scale showed one item with kappa above 0.81, four items between 0.61 and 0.80, ten items between 0.41 and 0.60, two items between 0.21 and 0.40 and only one item below 0.20. ICC was 0.928 (95% CI 0.877–0.958). Kendall’s tau-b ranged from 0.450 to 0.617 (*p* < 0.001).

**Conclusions:**

The newly developed scale represents a valid and reliable measure for assessing experiences of stigma among patients receiving care within mental health services. The scale has provided initial evidence of being specifically tailored for individuals with psychotic and bipolar disorders. However, the factorial structure of the scale should be replicated through a confirmatory factor analysis on a larger sample of individuals with these conditions.

## Introduction

Provider-based stigma within mental health services refers to the negative attitudes and behaviours that mental health providers exhibit towards individuals seeking mental health services. This type of stigma can take many forms, such as labelling, blaming or stereotyping patients (Pescosolido and Martin, 2015). The main driver of provider-based stigma is the societal stigma surrounding mental disorders. These conditions are often stigmatized, and mental health providers – as other members of the general population – may hold negative attitudes and beliefs, leading them to behave in stigmatizing ways towards their patients (Schulze, 2007). This type of stigma can have severe consequences for patients, including discouraging them from seeking help (Harangozo *et al.*, 2014), causing them to feel ashamed or embarrassed about their mental health problems (Gronholm *et al.*, 2017) and ultimately worsening their mental health outcomes (Corrigan and Watson, 2002).

The initial studies that investigated stigma within mental health services from the users’ perspective used qualitative methodologies, such as focus groups or open interviews, and found that certain relational modalities and intervention approaches employed by mental health professionals were perceived as stigmatizing by patients with severe mental disorders (Pinfold *et al.*, 2005; Schulze and Angermeyer, 2003). Several quantitative studies have subsequently revealed that at least one-quarter of individuals with mental disorders report experiences of discrimination within mental health services (Royal College of Psychiatrists, 2002; Walter, 1998). More recent studies have reported that the frequency of discrimination experienced by patients within mental health services ranges from 16% to 44% (Corker *et al.*, 2016; Gabbidon *et al.*, 2014; Harangozo *et al.*, 2014; Lasalvia *et al.*, 2013; Thornicroft *et al.*, 2014).

Provider-based stigma in mental health services, however, has received relatively little specific attention. While there is some research on this topic, the body of literature is relatively small compared to other areas of stigma research. One possible reason for the lack of research on provider-based stigma is that it can be difficult to measure and study. Provider-based stigma is a multidimensional construct that can involve attitudes, beliefs and behaviours of mental health providers, as well as the experiences and perceptions of individuals receiving care. This complexity can make it challenging to develop standardized measures and study designs that can capture the full range of provider-based stigma. Another possible reason for the under-researched nature of provider-based stigma is that mental health providers themselves may be hesitant to acknowledge their own biases and thus engage in research related to provider-based stigma. Despite these challenges, there is a growing recognition of the importance of understanding and addressing provider-based stigma in mental health services (Jauch *et al.*, 2023).

Recent research efforts have focused on developing standardized scales and tools to measure provider-based stigma in mental health services. These scales generally assess attitudes, beliefs and/or behavioural intentions of mental health providers towards individuals with mental illness. For example, the Opening Mind Scale for Health Care Providers (OMS-HC) (Kassam *et al.*, 2012) is a 20-item questionnaire developed to measure the stigma of mental illness among healthcare providers. The OMS-HC aims to capture attitudes, beliefs and behaviours related to stigma and discrimination towards people with mental health problems. Another measure that has been developed to assess provider-based stigma in mental health settings is the Mental Health Provider Stigma Inventory (MHPSI) (Kennedy *et al.*, 2017). The MHPSI is a 24-item scale that assesses mental health providers’ attitudes and behaviours towards individuals with mental illness and the influence of co-workers’ relationships on stigmatizing attitudes and behaviours. More recently, the Mental Health Provider Self-Assessment of Stigma Scale (Charles and Bentley, 2018) has been developed, a 20-item reliable self-rated scale for measuring attitudes and behavioural intentions that mental health providers may have toward one’s own service users.

In addition to these scales designed for mental healthcare providers, qualitative research has also been conducted to explore the experiences and perceptions of individuals receiving mental health care. These studies have provided valuable insights into the impact of provider-based stigma on the quality of care and outcomes for individuals with mental illness (Valery *et al.*, 2022). There is, however, less quantitative research on stigma experienced by people receiving care within mental health services. This is probably due to the lack of available assessment measures specifically designed to capture experiences of stigma by mental health service users themselves in their everyday interactions with mental health providers.

To fill this gap, we developed a new empirically derived, experience-based standardized scale for measuring experiences of stigma within mental health settings as perceived by people receiving mental health care themselves. The scale was developed by our research team together with the members of a mental health service users association, by adopting a participatory research approach (Trivedi and Wykes, 2002). In the context of mental health services, participatory research aims to empower service users and incorporate their perspectives, knowledge and expertise into all stages of the research process (Syrett, 2011; Thornicroft and Rose, 2005). When developing a scale to assess experiences of stigma within mental health services, the involvement of mental health service users may be crucial, as they can provide valuable input in shaping the content, wording and structure of the scale; contribute to identifying the key domains of stigma to be included in the scale; shed light on the specific aspects of stigma that are particularly impactful within mental health service; and ensure that the scale captures the nuances and dimensions of stigma that are most relevant to them. This paper aims to present the process that has led to the scale development and the psychometric evaluation of the newly developed scale.

## Methods

### Item generation and pretesting

The scale was developed collaboratively and involved both mental health professionals and service users. Item generation took place during a series of seven well-structured focus group sessions held at the South-Verona Community Mental Health Centre. The group was led by four experienced clinicians with diverse backgrounds: a senior clinical psychologist (T.P.), psychiatric rehabilitation therapist (C.D.), a trainee psychiatrist (S.P.) and senior researcher (A.L.). The focus groups included eight mental health service users from the South-Verona Community Mental Health Centre (members of the users’ association ‘The Open Circle’) and seven service users from the Community Mental Health Centre of San Bonifacio, a small town located in the eastern part of the province of Verona. All mental health service users involved in the focus groups had an extensive history of at least 10 years of receiving mental health care. Their diagnoses primarily included bipolar disorder, schizophrenia and psychotic spectrum disorders. Importantly, all participants were in a recovery phase during the focus group sessions.

Conducted biweekly from January to April 2018, each focus group session lasted for two hours. To ensure a comprehensive collection of participants’ input, discussions during the focus group sessions were audio recorded. The discussions were conducted using a recovery-oriented approach, facilitating an open exchange of ideas and opinions between ‘experts by experience’ (the users) and ‘experts by science’ (the professionals). The participants demonstrated a remarkable grasp and awareness of the topic, emphasizing the pervasive nature of stigma and its significance in their lives.

The initial draft of the questionnaire was developed after a thorough review of existing instruments and an examination of the stigma literature, with a specific focus on ‘provider-based’ stigma within mental health services. This literature review was complemented by insights from users obtained during focus group sessions. The questionnaire was designed for self-administered use using a Likert scale with five response options.

During the sixth focus group session, the first draft of the questionnaire was discussed with participants. Each item’s introduction was collectively read and shared, and the users provided feedback suggesting potential additions, modifications or eliminations. Unclear items were rephrased and simplified based on their requests. Initially, four items were intended for reverse scoring; however, after the group expressed difficulty in understanding them, they were aligned with the other items.

Based on the participants’ feedback, a second draft of the questionnaire was prepared and subsequently completed by the participants themselves during the seventh focus group session. This pilot test aimed to identify and correct potential errors in interpretation, confusion and inappropriate responses. Participants were asked to review each item and provide feedback on its content, wording and overall suitability. No further modification requests were made during this session to confirm the final version of the questionnaire. The participants demonstrated good acceptability and comprehension of the instrument, with an average completion time of approximately 15 min. In addition, a section on socio-demographic variables and other aspects related to diagnosis and treatment was added to the final version of the questionnaire.

### The questionnaire

The scale – Mental Health Service Stigma Assessment Questionnaire (MHS-SAQ) – was designed to measure experiences of stigmatization among people with mental disorders when interacting with mental health staff. The initial version of the scale consisted of 24 statements that capture specific aspects of experienced stigma within mental health services. Participants are asked to rate their agreement with each statement on a 4-point Likert scale, 0 = never, 1 = rarely, 2 = sometimes and 3 = often; a ‘not applicable’ option is also available. No items are reverse coded. The items are designed to cover a range of situations and scenarios commonly encountered within mental health services, including interactions with healthcare professionals, access to treatment options and perceptions of being treated differently because of one’s mental health condition. The scale also includes items that explore the emotional impact of experienced stigma and participants’ perceptions of their rights being violated. The wording and phrasing of the items were carefully crafted to ensure clarity and avoid ambiguity, with input from experts in stigma research and mental health service delivery.

A mean total score is calculated, with a higher score indicating higher experienced discrimination. The scale was originally developed in Italian; an English translation of the original Italian scale is given in the accompanying Appendix.

### Design

This study was cross-sectional, with participants being interviewed at one point in time. A subsample of participants also completed the scale again 14–20 days following initial administration to establish the test–retest reliability. Alongside the scale, participants also completed an additional measure, DISC-Ultra Short (DISCUS), a self-rated 11-item scale, which represents the short version of the DISC-12 (Brohan *et al.*, 2013), a well-established tool specifically designed to capture experiences of discrimination among people with mental disorders. By including the DISCUS in the study, we aimed to establish concurrent validity by examining the correlation between the new scale and an established measure of discrimination; DISCUS has proved to be a valid and reliable measure (Bakolis *et al.*, 2019), also in its Italian version (Lasalvia *et al.*, 2023). Data collection was performed between April and November 2022.

### Participants

The scale was administered by the research staff to a convenience sample of patients with the full range of mental disorders that at the time of data collection were all receiving care by public mental health services. The sample was recruited from different settings, including outpatient clinics, day care facilities and community-based support programs. The inclusion criteria encompassed patients aged 18 and above who were currently in contact with mental health services and had received mental health care for at least the preceding year. People admitted to in-patient units were not included in the study. Participants were provided with clear instructions on how to complete the scale and were assured of the confidentiality and anonymity of their responses. The scale was administered using a self-report questionnaire format, allowing participants to respond independently and at their own pace. All participants provided written informed consent. The study was performed in line with the principles of the Declaration of Helsinki. Approval was granted by the Ethics Committee of the Provinces of Verona and Rovigo (approval No. 23696, 11 April 2022).

### Statistical analysis

Psychometric properties were established by performing construct validity, reliability, precision, acceptability and feasibility. All analyses were performed with Stata 17 for Windows.

#### Construct validity

Construct validity was established by conducting an exploratory factor analysis based on the principal component factoring with Promax rotations. Kaiser–Meyer–Olkin’s (KMO) measure of sampling adequacy and Bartlett’s test of sphericity were estimated to explore the model’s adequacy. Factors with eigenvalues greater than one were retained. Only items with factor loadings >0.4 were considered in the final model. The correlation with the Italian version of the DISCUS scale was also performed.

#### Reliability

The reliability was assessed by considering (1) consistency over subscales (internal consistency) and (2) consistency over time (test–retest reliability). The internal consistency was assessed using Cronbach’s *α* with a criterion of 0.70 for a good internal consistency (Cronbach, 1951). To assess test–retest reliability of items, weighted kappa coefficients were calculated with values >0.41 indicating a moderate agreement (Landis and Koch, 1977). A two-way mixed effect intraclass correlation coefficient (ICC) was used to calculate the test–retest reliability for the total mean score and the subscales. A criterion of 0.75 was used to indicate acceptable reliability (Koo and Li, 2016).

#### Precision

The precision (i.e., how well each item fits within the scale) was examined by Kendall’s tau-b coefficient. A correlation <0.30 was indicative of unacceptable fit (Furr, 2021).

#### Acceptability

In order to establish the extent to which the scale was acceptable for the target population, the following aspects were examined: (1) maximum endorsement frequencies (MEF) and (2) aggregate adjacent endorsement frequencies (AEF) (Furr, 2021). In considering MEF, the *n* (%) of respondents endorsing each response category was established. MEF > 80% in any category indicates that the item may need further consideration. AEF assesses the proportion of responses in two or more adjacent scale points of an item, where the criterion of >10% was considered acceptable.

#### Feasibility

The feasibility was assessed by registering the time taken to complete each questionnaire. More than 20 minutes was considered indicative of an unbearable participant burden. Finally, the percentage of patients who completed the questionnaire was calculated.

## Results

### Patients’ characteristics

Overall, 240 patients participated in the study; 56 (23.3%) also completed the retest assessment after 2 weeks. Socio-demographic and diagnostic characteristics of the overall sample are given in Table 1.

**Table 1.** Socio-demographics and clinical characteristics of the study sample (*n* = 240)

	*n* (%)
Gender	(14 missing)
Male	106 (46.9)
Female	120 (53.1)
Age at the onset	(25 missing)
≤20 y	62 (28.8)
21−30 y	62 (28.8)
31−40 y	41 (19.1)
>40 y	50 (23.3)
Education	(23 missing)
Up to secondary education	74 (34.0)
Tertiary education	115 (53.0)
Degree	28 (13.0)
Employment	(28 missing)
Unemployed	57 (26.9)
Employed	57 (26.9)
Student	11 (5.2)
Housewife	14 (6.6)
Retired	73 (34.4)
Marital status	(25 missing)
Single	129 (60.0)
Married	57 (26.5)
Divorced/Widowed	29 (13.5)
Know the diagnosis	(18 missing)
No	65 (29.3)
Yes	157 (70.7)
Reported diagnosis	(3 missing)
Psychosis	61 (39.6)
Anxiety disorder/OCD	20 (13.0)
Bipolar disorder	25 (16.2)
Personality disorder	13 (8.4)
Depression	35 (22.7)
Hospitalized for mental disorders	(16 missing)
No	79 (35.3)
Yes	145 (64.7)

In brief, the sample had a balanced gender composition; most of the sample possessed a tertiary education or a degree (66%), were not currently employed (73.1%) and did not have a stable marital relationship (73.5%). Most respondents had an illness onset under the age of 40 (76.7%) and reported that they were aware of their psychiatric diagnosis (70.7%), with the majority reporting a diagnosis of a non-psychotic disorder (60.4%).

### Scoring

The initial version of the scale consisted of 24 items; however, after analysing the frequency distributions among the 240 participants, six items were excluded because of the prevalence of responses being concentrated in the ‘0 = never’ category (‘I felt considered by the mental health service staff as if I had to lower my life expectations because I have a mental health problem’; ‘I felt considered by the mental health service staff as if I couldn’t give my contribution to society’; ‘I felt considered by the mental health service staff as if I was more dangerous than other people’; ‘I felt considered by the mental health service staff as if I was recognizable in appearance and/or behaviour’; ‘I felt treated by the mental health service staff in an impersonalized manner’; ‘I felt treated by the mental health service staff in a cold and detached way’). The final version of the questionnaire used in the validation study thus comprised 18 items; the frequency distribution of responses to these items is presented in Table 2.

**Table 2.** Response frequencies and percentages for the individual items of the questionnaire (*n* = 240)

	Never	Rarely	Sometimes	Often	Not applicable
	*n* (%)	*n* (%)	*n* (%)	*n* (%)	*n* (%)
1. Considered as if I couldn’t have friendships outside MH services	204 (85.0)	14 (5.8)	14 (5.8)	8 (3.3)	0 (0.0)
2. Considered as if I couldn’t have a love affair	201 (83.8)	18 (7.5)	17 (7.1)	3 (1.3)	1 (0.4)
3. Considered as if I couldn’t start a family or to have children	210 (87.5)	11 (4.6)	13 (5.4)	5 (2.1)	1 (0.4)
4. Considered as if I couldn’t have my own home or accommodation	210 (87.5)	14 (5.8)	10 (4.2)	4 (1.7)	4 (0.8)
5. Considered as if I couldn’t start or continue education	210 (87.5)	13 (5.4)	13 (5.4)	3 (1.3)	1 (0.4)
6. Considered as if I couldn’t search for or maintain a job	190 (79.2)	25 (10.4)	16 (6.7)	8 (3.3)	1 (0.4)
7. Felt treated without respect for my privacy	200 (83.3)	19 (7.9)	16 (6.7)	5 (2.1)	0 (0.0)
8. Heard unfair and offensive comments about people with MH problems	197 (82.1)	23 (9.6)	15 (6.3)	5 (2.1)	0 (0.0)
9. Felt violated in my physical integrity and personal safety	214 (89.2)	13 (5.4)	10 (4.2)	3 (1.3)	0 (0.0)
10. Felt considered responsible for my MH problems	198 (82.8)	16 (6.7)	19 (7.9)	6 (2.5)	0 (0.0)
11. Felt treated as if my MH problems had no chance of recovery	186 (77.5)	19 (7.9)	22 (9.2)	13 (5.4)	0 (0.0)
12. Felt treated in a paternalistic way	192 (80.0)	19 (7.9)	22 (9.2)	7 (2.9)	0 (0.0)
13. Felt treated in disrespectful and humiliating way	199 (82.9)	19 (7.9)	18 (7.5)	4 (1.7)	0 (0.0)
14. Felt treated with authoritarianism	191 (79.6)	19 (7.9)	21 (8.8)	9 (3.8)	0 (0.0)
15. Felt excluded from possibility to negotiate my pharmacotherapy	185 (77.4)	22 (9.2)	19 (7.9)	13 (5.4)	0 (0.0)
16. Felt excluded from possibility to choose rehabilitation activities	200 (83.3)	20 (8.3)	12 (5.0)	4 (1.7)	4 (1.7)
17. Felt considered as if I were not a normal person	196 (82.4)	18 (7.6)	9 (3.8)	15 (6.3)	2 (0.8)
18. Felt identified by my disorder	198 (83.2)	18 (7.6)	15 (6.3)	7 (2.9)	0 (0.0)

Missing values: item 10: *n* = 1, item 15: *n* = 1, item 17: *n* = 2, item 18: *n* = 2.

Overall, combining the responses given to the options ‘rarely’, ‘sometime’ and ‘often’ between 10.9% and 22.5% of participants reported negative experiences within mental health services. When examining the five most concerning areas, 22.5% felt that mental health staff treated them as if their mental health problems were incurable; 22.5% felt excluded by their treating psychiatrists when it came to discussing pharmacological therapy options; 20.5% felt that mental health staff treated them authoritatively; 20.4% mentioned feeling that mental health staff regarded them as incapable of searching for or maintaining employment due to their mental health problems; 20% felt that mental health staff treated them in a paternalistic manner.

### Construct validity

Eighteen items converged over a two-factor solution accounting for 56.4% of the variance (KMO 0.903; Bartlett’s test *p* < 0.001). The first factor, named ‘Dignity violation and personhood devaluation’, accounted for 47.7% of the variance, and it was composed of 11 items; the second factor, named ‘Perceived life restrictions and social exclusion’, accounted for 8.7% of the variance and it comprised 7 items (Table 3).

**Table 3.** Factor loadings from the exploratory factor analysis (principal component extraction; Promax rotations; factor loadings >0.4 were retained) for the questionnaire (*n* = 240)

Items	Factor 1	Factor 2	Communalities
1. Considered as if I couldn’t have friendships outside MH services	0.167	**0.575**	0.480
2. Considered as if I couldn’t have a love affair	−0.056	**0.866**	0.692
3. Considered as if I couldn’t start a family or to have children	−0.226	**0.932**	0.653
4. Considered as if I couldn’t have my own home or accommodation	−0.059	**0.800**	0.584
5. Considered as if I couldn’t start or continue education	0.227	**0.519**	0.470
6. Considered as if I couldn’t search for or maintain a job	0.132	**0.651**	0.551
7. Felt treated without respect for my privacy	**0.708**	0.042	0.540
8. Heard unfair and offensive comments about people with MH problems	**0.642**	0.063	0.467
9. Felt violated in my physical integrity and personal safety	**0.502**	0.230	0.451
10. Felt considered responsible for my MH problems	**0.744**	−0.087	0.479
11. Felt treated as if my MH problems had no chance of recovery	0.283	**0.505**	0.516
12. Felt treated in a paternalistic way	**0.641**	0.213	0.628
13. Felt treated in disrespectful and humiliating way	**0.780**	−0.088	0.530
14. Felt treated with authoritarianism	**0.846**	−0.064	0.650
15. Felt excluded from possibility to negotiate my pharmacotherapy	**0.634**	0.058	0.452
16. Felt excluded from possibility to choose rehabilitation activities	**0.839**	−0.092	0.614
17. Felt considered as if I were not a normal person	**0.791**	0.055	0.684
18. Felt identified by my disorder	**0.822**	0.022	0.700
Eigenvalue	8.6	1.6	–
% Variance explained	47.7	8.7	–

The correlation with the validated DISCUS was significant for the total score (*r* = 0.331, *p* = 0.008) and factor 1 (*r* = 0.346, *p* = 0.006), but no for factor 2 (*r* = 0.241, *p* = 0.057). However, it is worth noting that the correlation between the DISCUS and the new scale yielded an extremely high correlation coefficient (*r* = 0.972, *p* = 0.028) when analysed in the subsample of patients with psychosis.

### Reliability

The Cronbach’s alpha value for the total score was 0.934, indicating an excellent internal consistency. The alpha value for the items ranged from 0.927 to 0.932. By considering the two factors, the Cronbach’s alpha was 0.918 and 0.858, respectively (Table 4).

**Table 4.** Internal consistency for the total score and the factors (Cronbach’s alpha) for the questionnaire (*n* = 240)

	Cronbach’s alpha		
Items	Total score	Factor 1	Factor 2
1. Considered as if I couldn’t have friendships outside MH services	0.931		0.843
2. Considered as if I couldn’t have a love affair	0.930		0.827
3. Considered as if I couldn’t start a family or to have children	0.932		0.838
4. Considered as if I couldn’t have my own home or accommodation	0.932		0.838
5. Considered as if I couldn’t start or continue education	0.931		0.842
6. Considered as if I couldn’t search for or maintain a job	0.930		0.834
7. Felt treated without respect for my privacy	0.930	0.912	
8. Heard unfair/offensive comments about people with MH problems	0.930	0.914	
9. Felt violated in my physical integrity and personal safety	0.931	0.915	
10. Felt considered responsible for my MH problems	0.931	0.914	
11. Felt treated as if my MH problems had no chance of recovery	0.930		0.844
12. Felt treated in a paternalistic way	0.927	0.909	
13. Felt treated in disrespectful and humiliating way	0.930	0.912	
14. Felt treated with authoritarianism	0.928	0.907	
15. Felt excluded from possibility to negotiate my pharmacotherapy	0.931	0.916	
16. Felt excluded from possibility to choose rehabilitation activities	0.929	0.910	
17. Felt considered as if I were not a normal person	0.927	0.907	
18. Felt identified by my disorder	0.927	0.905	
All items	0.934	0.918	0.858

By considering test–retest reliability, one item in the questionnaire had a kappa value above 0.81 (excellent agreement), four items between 0.61 and 0.80 (good agreement), ten items between 0.41 and 0.60 (moderate agreement), two items between 0.21 and 0.40 (fair agreement) and one item below 0.20 (due to the presence of category ‘0 = Never’, which was chosen by 100% of retest sample at the first assessment) (Table 5).

**Table 5.** Test–retest reliability for the items (weighted Cohen’s kappa) for the questionnaire (*n* = 56)

		Weighted Cohen’s kappa	
Items	Raw % agreement	Value	Test–retest reliability^a^
1. Considered as if I couldn’t have friendships outside MH services	92.9%	0.653	Substantial
2. Considered as if I couldn’t have a love affair	92.9%	0.569	Moderate
3. Considered as if I couldn’t start a family or to have children	94.7%	0.653	Substantial
4. Considered as if I couldn’t have my own home or accommodation	92.9%	0.522	Moderate
5. Considered as if I couldn’t start or continue education	94.7%	0.481	Moderate
6. Considered as if I couldn’t search for or maintain a job	96.5%	0.789	Substantial
7. Felt treated without respect for my privacy	91.1%	0.524	Moderate
8. Heard unfair/offensive comments about people with MH problems	92.9%	0.780	Substantial
9. Felt violated in my physical integrity and personal safety	94.6%	0.000	^b^
10. Felt considered responsible for my MH problems	87.5%	0.362	Fair
11. Felt treated as if my MH problems had no chance of recovery	84.0%	0.425	Moderate
12. Felt treated in a paternalistic way	92.9%	0.492	Moderate
13. Felt treated in disrespectful and humiliating way	92.9%	0.517	Moderate
14. Felt treated with authoritarianism	94.7%	0.824	Almost perfect
15. Felt excluded from possibility to negotiate my pharmacotherapy	89.3%	0.596	Moderate
16. Felt excluded from possibility to choose rehabilitation activities	91.1%	0.409	Moderate
17. Felt considered as if I were not a normal person	91.1%	0.539	Moderate
18. Felt identified by my disorder	92.9%	0.378	Fair

a <0.21 slight; 0.21–0.40 fair; 0.41–0.60 moderate; 0.61–0.80 substantial; >0.80 almost perfect (Landis and Koch, 1977).

b In the retest sample, the frequency for the category ‘0 = Never’ was 100% at the test assessment.

Finally, based on the categorization proposed by Koo and Li (2016) (0.51–0.75 moderate; 0.76–0.90 good; and 0.91–1 excellent), ICC calculated for the questionnaire showed that the total mean score (0.928, 95% CI 0.877–0.958) and factor 1 (0.918, 95% CI 0.860–0.952) had an excellent test–retest reliability, while factor 2 had a good reliability (0.882, 95% CI 0.860–0.952).

### Precision

The Kendall’s tau-b coefficients for the total scale ranged from 0.450 to 0.617 (*p* < 0.001), thus indicating that all items fit well with the score of the scale. Moreover, the two factors showed values ranging from 0.482 to 0.657 and from 0.536 to 0.696, respectively (Online Supplement, Table 1S).

### Acceptability

The MEF criterion was violated for the first response category (‘0 = never’) by 13 items in the overall sample (frequency ranging from 82.1% to 89.2%). However, upon analysing the frequency distribution of the response option ‘0 = never’ stratified by diagnostic categories, it emerged that in the subsamples of people with psychotic disorders and bipolar disorders, the MEF criterion was violated by only seven and ten items, respectively (details are provided in the Online Supplement, Table 2S). The AEF criterion was violated when considering the adjacent response categories ‘2 = sometimes’ and ‘3 = often’ for 11 items, ranging from 5.5% to 9.2%. Item 9 also violated the AEF criterion for the categories ‘1 = rarely’ and ‘2 = sometimes’ (9.6%).

### Feasibility

The mean completion times was about 10 (SD 5) minutes. Only 13 patients (5.1%) refused to fill in the scale.

### Difference of the questionnaire score by socio-demographics and clinical variables

Table 6 reports differences in the total score of the questionnaire by socio-demographics and clinical variables.

**Table 6.** Differences in total score for socio-demographics and clinical characteristics (*n* = 240)

	Total score mean (SD)	Test	*p*-value
Gender	(14 missing)	Mann–Whitney	0.128
Male	0.31 (0.51)		
Female	0.24 (0.45)		
Age at the onset	(25 missing)	Kruskal–Wallis	<0.001
≤20 y	0.45 (0.58)		
21−30 y	0.23 (0.47)		
31−40 y	0.33 (0.54)		
>40 y	0.07 (0.18)		
Education	(23 missing)	Kruskal–Wallis	0.122
Up to secondary education	0.32 (0.51)		
Tertiary education	0.28 (0.50)		
Degree	0.13 (0.30)		
Employment	(28 missing)	Kruskal–Wallis	0.010
Unemployed	0.36 (0.50)		
Employed	0.16 (0.43)		
Student	0.30 (0.52)		
Housewife	0.19 (0.56)		
Retired	0.29 (0.47)		
Marital status	(25 missing)	Kruskal–Wallis	<0.001
Single	0.37 (0.54)		
Married	0.10 (0.32)		
Divorced/Widowed	0.18 (0.37)		
Know the diagnosis	(18 missing)	Mann–Whitney	0.891
No	0.23 (0.41)		
Yes	0.29 (0.51)		
Reported diagnosis		Kruskal–Wallis	0.045
Psychosis	0.42 (0.59)		
Anxiety disorder/OCD	0.15 (0.36)		
Bipolar disorder	0.28 (0.59)		
Personality disorder	0.28 (0.49)		
Depression	0.14 (0.28)		
Hospitalized	(16 missing)	Mann–Whitney	<0.001
No	0.14 (0.34)		
Yes	0.33 (0.53)		

As shown in the table, patients who are younger, unemployed, single and report a diagnosis of psychosis, along with previous experiences of hospitalization, exhibited significantly higher total scores on the questionnaire. An overlapping pattern also emerged for the two-factor scores (see the Online Supplement, Tables 3S and 4S).

## Discussion

This study contributes to the field of mental health stigma research by providing a new standardized scale, the MHS-SAQ, that measures experiences of stigmatization within mental health settings. This scale fills a gap in the literature by providing a standardized measure that considers experiences of stigma within mental health services as perceived by individuals receiving mental health care themselves.

The main strength of this study is its participatory research approach. By engaging mental health service users as active participants in the research process, the resulting scale is more likely to accurately capture their experiences of stigma within mental health services and to ensure that the scale’s wording and phrasing are clear, understandable and resonate with their experiences. Moreover, this approach fosters empowerment, collaboration and a sense of ownership among service users, leading to more meaningful and impactful research outcome.

The psychometric evaluation conducted in this study shows that the 18-item MHS-SAQ, with its two-factor structure, is a reliable and valid self-report measure for assessing experiences of stigma within mental health services. The construct validity of the scale was established through exploratory factor analysis, which revealed two meaningful factors, ‘Dignity violation and personhood devaluation’ and ‘Perceived life restrictions and social exclusion’. The first factor emphasizes the experiences of feeling disrespected, violated, excluded and devalued in mental health services; it highlights the importance of dignity and personhood in the context of mental health care and the impact of these experiences on individuals’ well-being and sense of self. The second factor reflects the experiences of mental health service users of feeling excluded and limited in various aspects of their lives due to the way they are considered by mental health service staff; it highlights the restrictions and perceived barriers they face in forming relationships, pursuing personal goals such as starting a family or having a home and accessing educational or employment opportunities. Additionally, it acknowledges the perception that their mental health problems are seen as limiting their chances of recovery.

The scale demonstrated excellent internal consistency (as measured by Cronbach’s alpha) and acceptable test–retest reliability (as measured by kappa and ICCs). The scale demonstrated significant correlation with the DISCUS, supporting concurrent validity; the correlation with the DISCUS was particularly high in the subsample of people with psychosis, thus suggesting that the scale performs better on this specific population. The precision demonstrates that all items fit well with the scores of the questionnaire. The acceptability assessed by MEF was violated in a minimal number of items by considering the subsamples of people with psychotic and bipolar disorders, suggesting that the newly developed questionnaire exhibits a specific sensitivity in capturing experiences of discrimination within mental health services among individuals with more severe psychiatric conditions. Finally, the scale was completed within 10 minutes by most participants, thus proving to be a feasible instrument.

Notably, higher scores were observed among respondents who exhibited a distinct clinical profile, characterized by being younger, unmarried, unemployed, diagnosed with psychosis and having previous hospitalizations for mental health issues. These findings confirm that the scale may be specifically suited for individuals with severe mental disorders.

Finally, the data collected using the MHS-SAQ provides insights into various aspects concerning mental illness stigma within mental health services. Overall, our study supports existing literature (Jauch *et al.*, 2023) by confirming that mental illness stigma is indeed a significant issue within these services. Between 11% and 22.5% of participants reported experiencing unfair treatment by their healthcare providers. Furthermore, our study offers specific information on areas of particular concern. First, it is concerning that almost a quarter of participants felt stigmatized by mental health staff who considered their mental health problems incurable. This perpetuates the belief that recovery is unlikely, leading to demoralization among individuals seeking help and potentially hindering their willingness to seek assistance and access appropriate treatments. Moreover, almost a quarter of participants reported feeling excluded by their treating psychiatrists during discussions about pharmacological therapy options. This highlights a significant communication gap that inhibits shared decision-making and informed choices, ultimately undermining the quality of care provided (Drake *et al.*, 2009). Another troubling finding is that 20.5% of participants experienced authoritative treatment from mental health staff, which contradicts the patient-centred care model endorsed in the existing literature (Boardman and Dave, 2020). Addressing this issue is crucial as fostering a collaborative therapeutic relationship between mental health professionals and patients is essential for achieving effective treatment outcomes. Similarly, the fact that 20.4% of participants felt regarded as incapable of seeking or maintaining employment due to their mental health problems signifies the perpetuation of stigma within mental health services. Such perceptions reinforce societal biases and further hinder successful integration into the workforce for individuals already facing challenges. Furthermore, 20% of participants reported being treated in a paternalistic manner by mental health staff, highlighting the need for a more compassionate and patient-centric approach. Encouraging patient empowerment, shared decision-making and autonomy in treatment choices are essential in mitigating mental illness stigma and fostering positive therapeutic alliances.

### Strengths and limitations

Strengths of the study include the participatory research approach used in scale development, which incorporated the perspectives and expertise of mental health service users. The scale underwent rigorous psychometric evaluation, including assessments of construct validity, reliability, precision, acceptability and feasibility. The sample included patients with various mental disorders recruited from different mental health settings. Among limitations, it should be acknowledged that the sample addressed in this study is a convenient self-selected sample that may not be representative of all those in contact with mental health services. Further, nearly one-third of the participants either did not report their diagnosis or indicated that they did not know their diagnosis. To maintain anonymity and confidentiality, we relied on patients’ self-reports. While we acknowledge that this approach may potentially affect the clarity of our target population, we prioritized the principle of safeguarding participants’ anonymity. Moreover, this study was conducted in Italy, which may limit the generalizability of the findings to other cultural contexts.

### Implication and future research

The findings of this study have implications for mental health service delivery and research. The development of the MHS-SAQ provides a valid and reliable tool for assessing experiences of stigma within mental health services from the perspective of individuals receiving care. By understanding and addressing provider-based stigma, mental health service providers can create a more supportive and inclusive environment, which may positively impact patients’ help-seeking behaviour, treatment engagement and mental health outcomes.

As the scale has shown initial evidence of being specifically tailored for individuals with psychotic and bipolar disorders, a confirmatory factor analysis should be conducted on a larger sample of individuals with these conditions. Further research is also needed to validate the scale and its factorial structure in diverse geographical and cultural contexts and different treatment settings. Conduction studies with larger samples and incorporating longitudinal designs will also be crucial to assess the scale’s sensitivity to change over time.

## Conclusions

In conclusion, the stigma encountered by patients within mental health services poses a notable yet insufficiently explored issue. It is crucial for mental health professionals to recognize and address their personal biases and beliefs surrounding mental illness. This will help create a safe and supportive environment for patients. By tackling and reducing the stigma originating from healthcare providers, we can guarantee that every individual receives the mental health services that they require and are entitled to. The standardized scale presented in this study is expected to enable a comprehensive assessment of mental illness stigma within mental health services and facilitate the implementation of intervention studies aimed at mitigating this relevant problem.

## Supporting information

Lasalvia et al. supplementary material Lasalvia et al. supplementary material

## Data Availability

Data will be available upon reasonable request.

## Supplementary material

The supplementary material for this article can be found at https://doi.org/10.1017/S2045796023000689.
