# Supplementary material for: Development and validation of a new standardized measure for assessing experiences of discrimination within mental health services. A participatory research project
Source: Epidemiol Psychiatr Sci. 2023 Sep 8;32:e54. doi: 10.1017/S2045796023000689 (PMC10539746; doi:10.1017/S2045796023000689)
Supplement: Lasalvia et al. supplementary material [file S2045796023000689sup001.docx]

**On-line Supplementary material**

**Table 1S.** Precision for the total score and the two factors’ scores (Kendall’s tau-b, p<0.001) (n=240)

|  | **Total score** | **Factor 1**  **Dignity violation and personhood devaluation** | **Factor 2**  **Perceived life restrictions and social exclusion** |
| --- | --- | --- | --- |
| 1. Considered as if I couldn't have friendships outside MH services | 0.521 |  | 0.606 |
| 2. Considered as if I couldn’t have a love affair | 0.518 |  | 0.630 |
| 3. Considered as if I couldn’t start a family or to have children | 0.455 |  | 0.552 |
| 4. Considered as if I couldn’t have my own home or accommodation | 0.450 |  | 0.536 |
| 5. Considered as if I couldn’t start or continue education | 0.469 |  | 0.539 |
| 6. Considered as if I couldn’t search for or maintain a job | 0.554 |  | 0.677 |
| 7. Felt treated without respect for my privacy | 0.536 | 0.581 |  |
| 8. Heard unfair/offensive comments about people with MH problems | 0.538 | 0.569 |  |
| 9. Felt violated in my physical integrity and personal safety | 0.455 | 0.482 |  |
| 10. Felt considered responsible for my MH problems | 0.524 | 0.564 |  |
| 11. Felt treated as if my MH problems had no chance of recovery | 0.585 |  | 0.696 |
| 12. Felt treated in a paternalistic way | 0.617 | 0.634 |  |
| 13. Felt treated in disrespectful and humiliating way | 0.533 | 0.588 |  |
| 14. Felt treated with authoritarianism | 0.605 | 0.657 |  |
| 15. Felt excluded from possibility to negotiate my pharmacotherapy | 0.574 | 0.618 |  |
| 16. Felt excluded from possibility to choose rehabilitation activities | 0.512 | 0.560 |  |
| 17. Felt considered as if I were not a normal person | 0.598 | 0.628 |  |
| 18. Felt identified by my disorder | 0.581 | 0.617 |  |

**Table 2S.** Frequency distribution of the response option “never” by diagnostic categories (n=240) (in bold are reported items no displaying a floor effect – MEF<80%)

|  | **Psychosis**  **%** | **Anxiety disorder/ OCD**  **%** | **Bipolar disorder**  **%** | **Personality disorder**  **%** | **Depression**  **%** | **Diagnosis unknown**  **%** |
| --- | --- | --- | --- | --- | --- | --- |
| 1. Considered as if I couldn't have friendships outside MH services | 82.0 | 90.0 | 88.0 | 100.0 | 94.3 | 83.1 |
| 2. Considered as if I couldn’t have a love affair | 83.6 | 89.5 | **80.0** | 92.3 | 85.7 | 87.7 |
| 3. Considered as if I couldn’t start a family or to have children | 85.2 | 90.0 | **80.0** | 92.3 | 94.3 | 93.8 |
| 4. Considered as if I couldn’t have my own home | 85.2 | 90.0 | 84.0 | 92.3 | 88.2 | 92.2 |
| 5. Considered as if I couldn’t start or continue education | 85.2 | 85.0 | 87.5 | 100.0 | 97.1 | 87.7 |
| 6. Considered as if I couldn’t search for or maintain a job | **70.5** | 80.0 | 88.0 | 92.3 | 88.6 | 82.8 |
| 7. Felt treated without respect for my privacy | **73.8** | 90.0 | 92.0 | 84.6 | 91.4 | 86.2 |
| 8. Heard offensive comments about people with MH problems | **75.4** | 90.0 | 84.0 | 92.3 | 94.3 | **80.0** |
| 9. Felt violated in my physical integrity and personal safety | 82.0 | 95.0 | 92.0 | 92.3 | 94.3 | 93.8 |
| 10. Felt considered responsible for my MH problems | 80.3 | 90.0 | **76.0** | **76.9** | 88.2 | 81.5 |
| 11. Felt treated as if my MH problems had no chance of recovery | **72.1** | 85.0 | **76.0** | 69.2 | 85.7 | 81.5 |
| 12. Felt treated in a paternalistic way | **77.0** | 85.0 | **80.0** | **76.9** | 91.4 | 81.5 |
| 13. Felt treated in disrespectful and humiliating way | **75.4** | 95.0 | 88.0 | 69.2 | 88.6 | 89.2 |
| 14. Felt treated with authoritarianism | **73.8** | 85.0 | **80.0** | 84.6 | 88.6 | 81.5 |
| 15. Felt excluded from possibility to negotiate my pharmacotherapy | **70.5** | 85.0 | **72.0** | 84.6 | 82.9 | 81.3 |
| 16. Felt excluded from possibility to choose rehabilitation activities | **75.4** | 90.0 | 87.5 | 91.7 | 90.9 | 86.2 |
| 17. Felt considered as if I were not a normal person | **78.3** | 90.0 | 88.0 | **69.2** | 94.1 | 87.7 |
| 18. Felt identified by my disorder | **73.8** | 90.0 | 87.5 | 84.6 | 97.1 | 87.5 |

missing values: item 10 n=1, item 15 n=1, item 17 n=2, item 18 n=2

**Table 3S.** Differences in Factor 1 score (“Dignity violation and personhood devaluation”) for socio-demographics and clinical characteristics (n=240)

|  | **FACTOR 1**  **Mean (sd)** | **Test** | **p-value** |
| --- | --- | --- | --- |
| **Gender**  Male  Female | (14 missing)  0.33 (0.60)  0.23 (0.46) | Mann-Whitney | 0.137 |
| **Age at the onset**  <=20 yrs.  21-30 yrs.  31-40 yrs.  >40 yrs. | (25 missing)  0.51 (0.70)  0.20 (0.44)  0.33 (0.53)  0.08 (0.26) | Kruskal-Wallis | <0.001 |
| **Education**  Up to secondary education  Tertiary education  Degree | (23 missing)  0.31 (0.51)  0.31 (0.59)  0.14 (0.31) | Kruskal-Wallis | 0.219 |
| **Employment**  Unemployed  Employed  Student  Housewife  Retired | (28 missing)  0.39 (0.62)  0.15 (0.43)  0.31 (0.55)  0.17 (0.56)  0.29 (0.50) | Kruskal-Wallis | 0.030 |
| **Marital status**  Single  Married  Divorced/Widowed | (25 missing)  0.39 (0.61)  0.08 (0.30)  0.19 (0.38) | Kruskal-Wallis | <0.001 |
| **Know the diagnosis**  No  Yes | (18 missing)  0.24 (0.46)  0.30 (0.56) | Mann-Whitney | 0.920 |
| **Reported diagnosis**  Psychosis  Anxiety disorder/OCD  Bipolar disorder  Personality disorder  Depression | (3 missing)  0.45 (0.68)  0.13 (0.34)  0.26 (0.58)  0.36 (0.67)  0.15 (0.30) | Kruskal-Wallis | 0.092 |
| **Hospitalised**  No  Yes | (16 missing)  0.15 (0.38)  0.35 (0.59) | Mann-Whitney | <0.001 |

**Table 4S.** Differences in Factor 2 score (“Perceived life restrictions and social exclusion”) for socio-demographics and clinical characteristics (n=240)

|  | **FACTOR 2**  **Mean (sd)** | **Test** | **p-value** |
| --- | --- | --- | --- |
| **Gender**  Male  Female | (14 missing)  0.26 (0.46)  0.24 (0.52) | Mann-Whitney | 0.132 |
| **Age at the onset**  <=20 yrs.  21-30 yrs.  31-40 yrs.  >40 yrs. | (25 missing)  0.35 (0.55)  0.27 (0.54)  0.33 (0.58)  0.05 (0.13) | Kruskal-Wallis | 0.006 |
| **Education**  Up to secondary education  Tertiary education  Degree | (23 missing)  0.34 (0.57)  0.23 (0.48)  0.13 (0.34) | Kruskal-Wallis | 0.127 |
| **Employment**  Unemployed  Employed  Student  Housewife  Retired | (28 missing)  0.30 (0.48)  0.16 (0.44)  0.30 (0.50)  0.22 (0.57)  0.30 (0.55) | Kruskal-Wallis | 0.174 |
| **Marital status**  Single  Married  Divorced/widowed | (25 missing)  0.34 (0.56)  0.12 (0.37)  0.18 (0.38) | Kruskal-Wallis | 0.002 |
| **Know the diagnosis**  No  Yes | (18 missing)  0.21 (0.38)  0.28 (0.53) | Mann-Whitney | 0.925 |
| **Reported diagnosis**  Psychosis  Anxiety disorder/OCD  Bipolar disorder  Personality disorder  Depression | (3 missing)  0.38 (0.63)  0.19 (0.44)  0.33 (0.65)  0.15 (0.26)  0.14 (0.28) | Kruskal-Wallis | 0.284 |
| **Hospitalised**  No  Yes | (16 missing)  0.13 (0.33)  0.31 (0.55) | Mann-Whitney | 0.002 |
